# The role of phosphodiesterase 3 in endotoxin-induced acute kidney injury

**DOI:** 10.1186/1471-2334-9-80

**Published:** 2009-06-01

**Authors:** Won-Il Choi, Kun Young Kwon, Jeong Wook Seo, John Beagle, Deborah A Quinn, Charles A Hales

**Affiliations:** 1 Pulmonary Unit, Department of Internal Medicine, Dongsan Hospital, Keimyung University School of Medicine, Daegu, Korea; 2 School of Medicine & Institute for Medical Genetics, Keimyung University, Daegu, Korea; 3 Pulmonary/Critical Care Unit, Department of Medicine, Massachusetts General Hospital and Harvard Medical School, Boston, MA; 4 Department of Pathology, Dongsan Hospital, Keimyung University School of Medicine, Daegu, Korea; 5 Department of Pathology, Seoul National University Hospital, Seoul National University College of Medicine, Seoul, Korea

## Abstract

**Background:**

Acute kidney injury frequently accompanies sepsis. Endotoxin is known to reduce tissue levels of cAMP and low levels of cAMP have been associated with renal injury. We, therefore, hypothesized that endotoxin induced renal injury by activating phosphodiesterase 3 (PDE3) which metabolizes cAMP and that amrinone an inhibitor of PDE3 would prevent the renal injury.

**Methods:**

Animals were divided into three groups (n = 7/group): 1) Control (0.9% NaCl infusion without LPS); 2) LPS (0.9% NaCl infusion with LPS); 3) Amrinone+LPS (Amrinone infusion with LPS). Either lipopolysaccharide (LPS) or vehicle was injected via the jugular vein and the rats followed for 3 hours. We explored the expression of PDE3 isoenzymes and the concentrations of cAMP in the tissue.

**Results:**

The PDE3B gene but not PDE3A was upregulated in the kidney of LPS group. Immunohistochemistry also showed that PDE3B was expressed in the distal tubule in the controls and LPS caused PDE3B expression in the proximal as well. However, PDE3A was not expressed in the kidney either in the control or LPS treated groups. Tissue level of cAMP was decreased after LPS and was associated with an increase in blood urea nitrogen, creatinine, ultrastructural proximal tubular changes, and expression of inducible nitric oxide synthase (iNOS) in the endotoxemic kidney. In septic animals the phosphodiesterase 3 inhibitor, amrinone, preserved the tissue cAMP level, renal structural changes, and attenuated the increased blood urea nitrogen, creatinine, and iNOS expression in the kidney.

**Conclusion:**

These findings suggest a significant role for PDE3B as an important mediator of LPS-induced acute kidney injury.

## Background

Acute renal failure (ARF) frequently occurs in the systemic inflammatory response syndrome. In patients with the combination of sepsis and ARF mortality is as high as 70% [1,2]. During endotoxemia, injury to the renal tubule has been considered to be one of the most important sites undergoing renal injury [3].

Lipopolysaccharide (LPS) can cause a decrease in tissue cAMP concentration [4]. An increase in cellular cAMP protects hypoxemic induced endothelial injury by preserving its barrier function [5], and improves post-ischemic recovery in the liver and kidney [6,7].

Tissue Cyclic-3',5'-adenosine monophosphate (cAMP) levels are determined by the balance between activities of the synthesizing enzyme and the catabolizing enzymes, such as the cyclic 3',5'-nucleotide phosphodiesterases (PDE) that hydrolyze the 3'-phosphoester bond of cAMP to its biologically inactive noncyclic nucleotides 5'-AMP [8]. Type 3 phsophodiesterase inhibitors (PDEIs) increase intracellular cAMP by inhibition of phosphodiesterase enzyme [9]. There are two isogenes, PDE3A and PDE3B, which differ in their distribution in organ and tissue [10]. The PDE3B gene has been shown to be present in the urogenito-epithelium [10].

Lipopolysaccharide (LPS) is one of the important activators of iNOS (inducible nitric oxide synthase) [11]. Specific iNOS inhibitors are effective in attenuating kidney injury with ischemia/reperfusion [12] and with LPS induced renal dysfunction [13,14]. Although the PDE3 inhibitor, amirinone, reduces LPS stimulated iNOS activiation *in vitro *[15], its effects on *in vivo *endotoxemia are unknown.

The aim of the present study was to examine renal tubular morphologic changes and renal function occurring in LPS-induced acute kidney injury in relation to the effect of LPS on expression of PDE 3A and 3B in the kidney. It was our hypothesis that LPS would cause renal injury via PDE3 activation causing catabolism of cAMP leading in turn to upregulation of iNOS and that the specific PDE3 inhibitor, amrinone [16], would prevent activation of the cAMP-iNOS pathway with subsequent kidney injury.

## Methods

### Animal Preparation

Male Sprague Dawley rats weighing between 180 and 230 gm were anesthetized with 80 mg/kg of ketamine and 5 mg/kg of diazepam intraperitoneally. This protocol was approved by the Keimyung University School of Medicine and Massachusetts General Hospital Committee on Animal Research.

### Experimental Protocol

Silastic (0.012 inch I.D, 0.025 inch O.D) catheters were placed in the right jugular vein. Endotoxemia was induced by administration of *E. coli *LPS (1 mg/kg; *E. coli *0111:B4, Sigma, St. Luois, MO), single bolus injection through the jugular vein. The LPS dose was selected from the dose used in other experiments to induce mild endotoxemia [17-22]. Animals were divided into three groups (n = 7/group): 1) Control (0.9% NaCl infusion without LPS); 2) LPS (0.9% NaCl infusion with LPS); 3) Amrinone+LPS (Amrinone infusion with LPS). In the amrinone treated groups, amrinone (Sigma, St. Louis, MO) was continuously infused for 3 h and 15 min at a dose of 40 μg/kg/min (1 ml/kg/h) [23]. LPS was injected 15 min after starting a continuous infusion of either 0.9% NaCl or amrinone. In the control and LPS groups, the same volume of 0.9% NaCl (1 ml/kg/h) was infused continuously. The kidneys were harvested for examination of histopathologic changes (n = 6/group), ultrastructural changes (n = 5/group), and renal tissue cAMP levels (n = 7/group).

### Hemodynamic Measurements

Silastic (0.012 inch I.D, 0.025 inch O.D) catheters were placed in the left carotid artery to monitor systemic artery pressure on a Gould recorder (Model R53400, Glen Burnie, MD) and it recorded at 30 minutes intervals.

### Reverse transcription-polymerase chain reaction

The expression of PDE3A, PDE3B, and GAPDH was analyzed by reverse transcription-polymerase chain reaction (RT-PCR). Total RNA was isolated by using TRIzole reagent. (Gibco BRL, Grand Island, NY). Five micrograms of total RNA from each specimen were used for complementary DNA synthesis. The PCR reaction was carried out using cDNA prepared using reverse transcriptase. The following protocol was used for the PCR: 95°C for 5 min and 35 cycles of 95°C for 30 s 50°C for 30 s and 72°C for 1 min and 30 s. This was followed by a final extension of 10 min at 65°C. PCR with specific forward and reverse regular or restriction tagged primers was used to amplify PDE transcripts. The PDE3A forward primer was CTG GCC AAC CTT CAG GAA TC and the reverse primer was GCC TCT TGG TTT CCC TTT CTC. The PDE3B forward primer was AAT CTT GGT CTG CCC ATC AGT CC and the reverse primer was TTC AGT GAG GTG GTG CAT TAG CTG [24]. Another PDE3A forward primer was CCG AAT TCC CTT ATC ATA ACA GAA TCC ACG CCA CT and reverse primer was GGG AAT TCG TGT TTC TTC AGG TCA GTA GCC [25]. Forward primer for a 528-bp fragment of the housekeeping gene glyceraldehyde-3-phosphate dehydrogenase (GAPDH) was ACC ACC ATG GAG AAG GCT GG and reverse primer was CTC AGT GTA GCC CAG GAT GC. RT-PCR results were quantified by densitometry using a Bio RAD imaging densitometer (Model G.S.-690) in conjunction with Molecular Analyst Software, Version 2.1 (Bio Rad laboratories, USA). Optical densities were expressed as arbitrary units.

### Blood Urea Nitrogen (BUN) and Serum Creatinine Determination

Blood samples were collected from the inferior vena cava to measure creatinine at the end of the experiment. Blood samples were centrifuged at 4°C for 15 min at 1200 × g in glass tubes, and the serum was stored at -80°C in polystyrene tubes until use. Estimation of BUN and creatinine concentration was performed using a creatinine analyzer (Beckman Instruments, Inc., Fullerton, CA).

### Tissue cAMP

Kidney tissue cAMP was measured by a spectrophotometric colorimetric 96 plate ELISA technique. Briefly, the renal cortex was immersed in liquid nitrogen, which was then homogenized in ice-cold 5% trichloroacetic-acid solution and centrifuged. The supernatant was used for the analysis of cAMP using an ELISA kit (R&D Systems, Minneapolis, MN).

### Histopathology and Immunohistochemistry

The kidneys were fixed in 10% buffered formalin for 24 h before processing and embedding into paraffin. Two sections were cut from each sample and stained with hematoxylin and eosin. The paraffin sections were cut to 5 μm in thickness, mounted on silane-coated glass slides, and stored for 1 h at 60°C. The slides were deparaffinized with xylene, three times, 5 min each, and were rehydrated with graded alcohols (100, 95, 70 and 50%) for 5 min, respectively. After washing with 0.01 M phosphate buffered saline (PBS) for 5 min, the sections were digested with Proteinase K (20 μg/ml) at room temperature for 20 min, and were washed twice with distilled water for 2 min each. The endogenous peroxidase activity was blocked with 3% hydrogen peroxide (H_2_O_2_) in PBS for 5 min; the slides were rinsed twice with PBS for 5 min. Sections for positive control were treated with 3% H_2_O_2 _and reacted with DNase I (1–2 U) at 37°C for 30 min, then washed twice with PBS. For negative controls, sections were covered with reaction buffer alone and incubated following same conditions. The sections were incubated 1.5 h with polyclonal antibodies against goat PDE3A and PDE3B (Abcam, Cambridge, UK) at a concentration of 10 μg/ml. For microscopic observation, the sections were counterstained lightly with hematoxylin for one min.

### Electron microscopy

The details for the analysis have been described elsewhere [26]. The cortical tissues were sliced into small pieces, and were fixed in periodate lysine paraformaldehyde (PLP) solution at 4°C overnight. After washing with 0.01 M PBS, the kidney tissues were immersed in graded concentrations of sucrose in PBS (10% for 1 h, 15% for 2 h, 20% for 4 h) at 4°C. Tissue fragments were post-fixed in cacodylate buffered 1% OsO4 for 1 h, and embedded in PolyBed mix resin (Poly/Bed 812, Polysciences Inc., USA). After trimming the PolyBed mix resin blocks, ultrathin sections (100 nm) were cut. Sections were examined using an electron microscope (Hitachi H-7100, Hitachi Co., Japan). Five representative areas of five samples per group were photographed. Two morphologic patterns, intracellular edema and mitochondrial integrity, were graded with a 5-point scale as follows [27]: 0, no abnormality; 1, mild lesions affecting 10% or less of kidney samples; 2, lesions affecting 25% of kidney samples; 3, lesions affecting 50% of kidney samples, and 4, lesions affecting 75% or more of kidney samples. These evaluations were performed in a blinded fashion.

### Immunoblot Analysis for PDE3A and inducible nitric oxide synthase

Kidneys from a different group of rats were removed and immediately frozen in liquid nitrogen 3 hours after LPS injection. Samples of isolated kidney were lysed in RIPA buffer (50 μM Tris-HCL [pH 7.5], 150 μM NaCl, 1% Triton X-100, 1% deoxycholate, and 0.1% sodium dodecyl sulfate) that contained 25 μg/ml aprotinin, 2 mM sodium orthovanadate, 25 μg/ml leupeptin, 2 mM phenylmethysulfonyl fluoride, and 50 mM sodium fluoride for 30 minutes on ice. Lysates were clarified by centrifugation at 15,500 × g for 15 minutes at 4°C. The protein concentration of the supernatant was measured with a Bio-Rad Protein Assay Kit. The supernatant was mixed with 2× sample buffer (114 mM Tris, 9% glycerol, 2.7% sodium dodecyl sulfate, 0.02% bromophenol blue, and 4.5% mercaptoehtanol), boiled for 5 minutes, subjected to 10% sodium dodecyl sulfate-polyacrylamide gel electrophoresis, and transferred onto polyvinylidine fluoride (PVDF) membrane (Bio-rad, Hercules, CA). Nonspecific binding sites were blocked by phosphate-buffered saline (PBS)-0.1% Tween 20 supplemented with 5% skim milk for 1 hour at room temperature. The membranes were incubated with anti-iNOS antibody (Santa Cruz Biotechnology, Santa Cruz, CA, 1:200) and anti-PDE3A antibody (Abcam, Cambridge, UK, 1:500) for overnight at 4°C. After extensive washing with three changes of PBS-0.1% Tween 20, the membranes were incubated with horseradish peroxidase-conjugated secondary for one hour. Immunoreactive proteins were visualized with an enhanced chemiluminescense detection system, followed by exposure to Kodak x-ray film (Eastman Kodak, Rochester, NY).

### Statistical Methods

Analysis was performed using Statview 4.5 (SAS Institute Inc., Cary, NC). All values were expressed as means ± standard error of mean. Analysis of variance (ANOVA) for comparison of the different groups was used with significance set at p < 0.05. A significant ANOVA was followed by a Scheffe-test for multiple comparisons between groups. Blood pressure was analyzed by two-way repeated measures analysis of variance and *post hoc *tests comparing group, time and their interactions, with a p < 0.05.

## Results

### Hemodynamics

**Figure 1:**
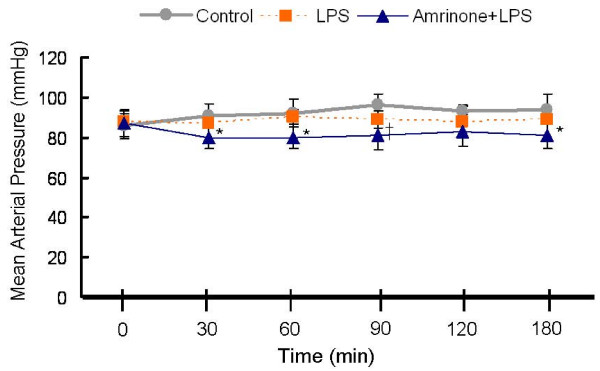
**Effects of Lipopolysaccharide (LPS) and type 3 phosphodiesterase (PDE3) inhibitor on mean arterial pressure (MAP) in rats (n = 7 per group)**. PDE3 inhibitor, amrinone, mildly reduced MAP. *P < 0.05 versus Control or LPS; ^†^*P *< 0.05 versus Control.

Endotoxin and the PDE inhibitor, both may have an effect on systemic hemodynamics. We monitored mean arterial pressure (MAP) continuously. At baseline (after anesthesia), MAP showed no differences among the groups (Figure 1). MAP did not change in the control and LPS groups throughout the experiment. However, in the amrinone+LPS group, MAP was mildly but significantly lower 30 min after starting of amrinone infusion compared with the two other groups. In the amrinone+LPS group, MAP remained above 80 mm Hg throughout the experiment, while the other 2 groups MAP stayed above 85 mm Hg.

### Expression of PDE3A and PDE3B in the kidney

**Figure 2:**
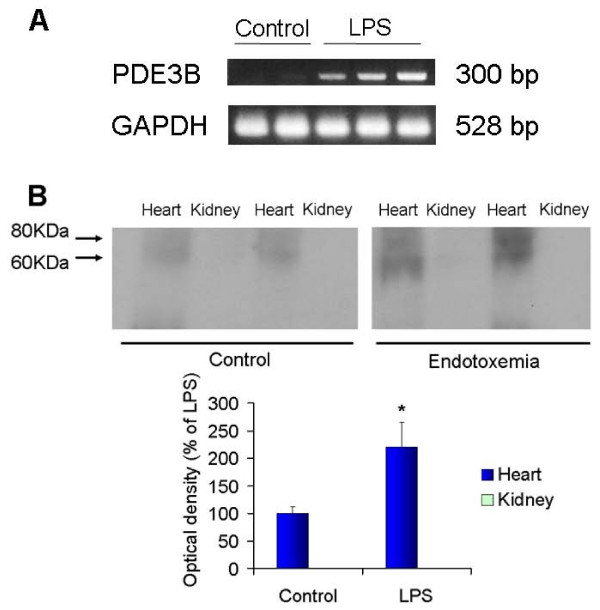
**A representative RT-PCR on total RNA from rat kidneys (A)**. A representative immunoblotting for PDE3A in the kidney and heart tissue extracts prepared from control and LPS treated group (B). LPS treatment induced 9-fold greater expression of PDE3B mRNA transcript than control. GAPDH was used as a loading control (A). PDE3A was not detected in the kidney tissue either with LPS or without LPS. However, PDE3A was detected in the heart tissue, and LPS caused an increase in its expression in the heart (B).

RT-PCR of kidney tissue was carried out to evaluate mRNA expression of PDE3 isoenzymes. RT-PCR yielded the expected size fragment, 300 base pair products, for the primer pair PDE3B. Although we used two differently designed fragments for the primer pairs of PDE3A, RT-PCR did not produce the expected sized fragments of PDE3A. LPS treatment induced PDE3B mRNA expression in kidney tissue; it was 9-fold greater than control (Figure 2A). To confirm no expression of the PDE3A enzyme in the kidney with or without LPS stimulation, we performed immunoblotting of PDE3A. Heart was used for positive control of PDE3A. PDE3A was expressed neither in the control kidney nor LPS treated, while it is expressed in the heart (Figure 2B). This result suggested that PDE3B gene but not PDE3A was stimulated by LPS in the kidney.

### Immunohistochemistry of PDE3

To confirm the RT-PCR results and evaluate structural localization of PDE3 protein expression in the kidney tissue, we performed immunohistochemistry of the two PDE3 isoenzymes.

**Figure 3:**
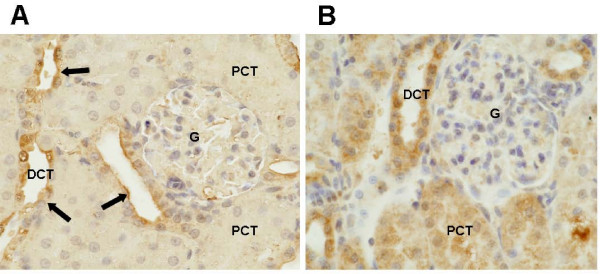
**PDE3B expression in a control kidney (A) and one treated with LPS (B)**. In the control group (A), immunohistochemical stain for PDE3B showed positive expression in the epithelial cells of the distal convoluted tubules (arrows). The glomerulus and proximal convoluted tubules showed no prominent expression for PDE3B. In the LPS group (B), both proximal and distal convoluted tubules showed strong expression for PDE3B (B). PCT: proximal convoluted tubule, DCT: distal convoluted tubule, G: glomerulus. Original magnification, ×200.

PDE3A was not detectable in both the control (n = 6) and LPS (n = 6) treated kidneys. However, PDE3B was expressed in the distal tubule in the control group in all six kidneys. In the LPS exposed rats, PDE3B is expressed on both the proximal and distal tubule in all six kidneys. PDE3B was not present in glomeruli. A typical staining pattern is demonstrated in Figure 3. These results confirmed that PDE3A was not expressed but PDE3B was expressed in the kidney tissue. The major activating site of PDE3B protein was the proximal tubule.

### Effect of LPS and amrinone on kidney tissue cAMP

**Figure 4:**
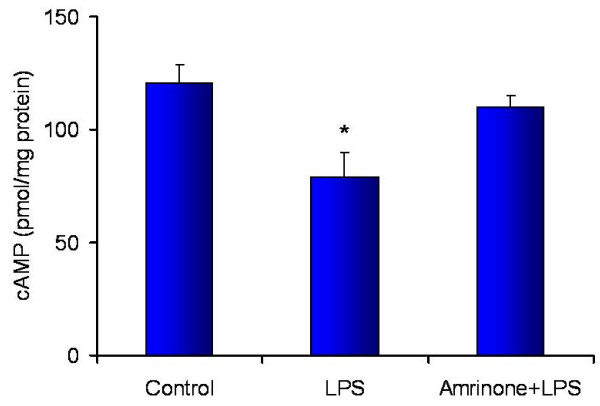
**cAMP concentration (as determined by ELISA) in renal cortex at the end of the experiment (n = 7/group)**. *P < 0.05 versus control or Amrinone+LPS.

There was a significant decrease in cAMP in LPS challenged kidney tissue at the end of the experiment (saline 120 ± 9 vs. LPS 79 ± 11 pmol/mg protein; n = 7/group; *P *< 0.05), a 35% reduction of cAMP level compared with baseline. Amrinone infusion prevented the fall in kidney tissue cAMP level leaving it comparable to control (110 ± 5 pmol/mg protein) (Figure 4). These results indicated that about 75% of the decreased cAMP levels in the LPS treated tissue was preserved by PDE3 inhibition.

### Blood urea nitrogen (BUN) and Creatinine

**Figure 5:**
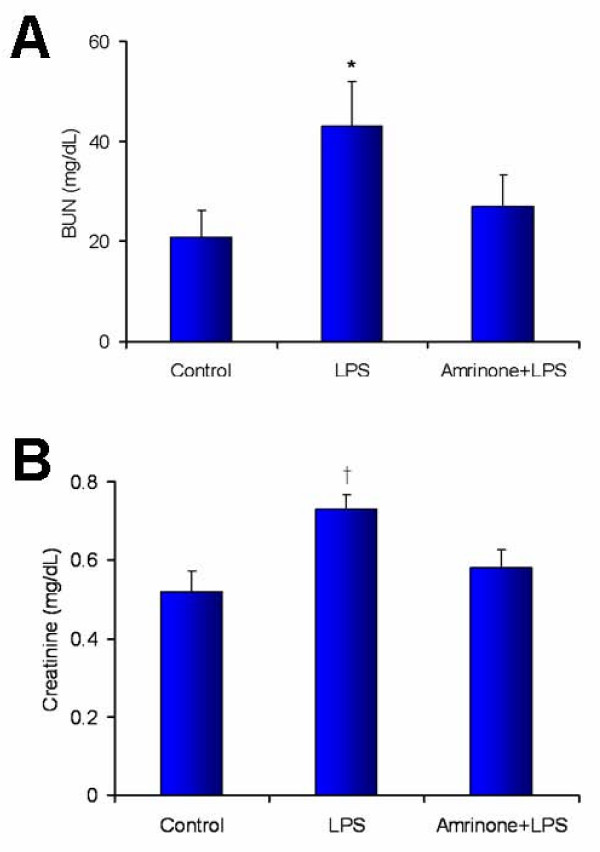
**Effects of LPS administration and amrinone infusion on serum BUN (A) and creatinine (B) (n = 7/group)**. *P < 0.05 versus control or Amrinone+LPS; ^†^*P *< 0.01 versus control or Amrinone+LPS.

Renal function was assessed 3 h after administration of LPS. Renal failure occurred at 3 h as evidenced by a rise in BUN (saline 21 ± 5 vs. LPS 43 ± 8 mg/dl; *P *< 0.05) and serum creatinine levels (saline 0.52 ± 0.05 vs. LPS 0.73 ± 0.04 mg/dl; n = 7/group; *P *< 0.01). The PDE3 inhibitor, amrinone, (40 μg/min/kg) significantly attenuated BUN (27 ± 6 mg/dl; *P *< 0.05) and creatinine levels (0.58 ± 0.04 mg/dl; *P *< 0.01) (Figure 5). This suggested that PDE3 inhibition attenuated endotoxin-induced renal failure.

### Electron Microscopy

**Figure 6:**
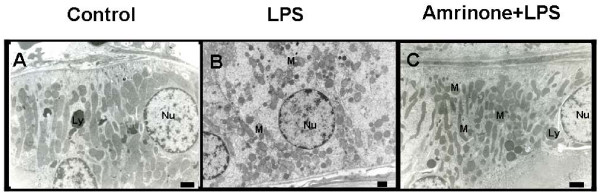
**Electron micrographs of the proximal tubule**. Control animal showed a normal tubule and normal appearance of mitochodria (A). Loss of polarity of mitochondria and increased perinuclear clear space occurred in the endotoxemic kidney (B). Amrinone infusion restored mitochondrial integrity and intracellular edema (C). Scale Bar = 5 μm. *Nu*, nucleus; *M*, mitochondria; *Ly*, lysosome.

Since light microscopic examination of renal tissue revealed normal histology, we investigated ultrastructural changes of the renal proximal tubule. In the control group, there was well preserved integrity of mitochondria without cellular edema. However, there was a substantial change in the mitochondria and in the intracellular edema in the proximal tubules in the LPS group (Figure 6). Amrinone attenuated the change of mitochondrial integrity and cellular edema. The result of the numerical scores and means are presented in Table 1.

**Table 1:** Electron microscopy changes in proximal tubules.

	Control	LPS	Amrinone+ LPS
Intracellular edema	0.5 ± 0.2	1.5 ± 0.3*	0.7 ± 0.2
Mitochondrial integrity	0.4 ± 0.2	1.7 ± 0.4*	0.8 ± 0.3

* *P *< 0.05 versus control or Amrinone+ LPS.

### Inducible Nitric Oxide Synthase (iNOS) Expression

**Figure 7:**
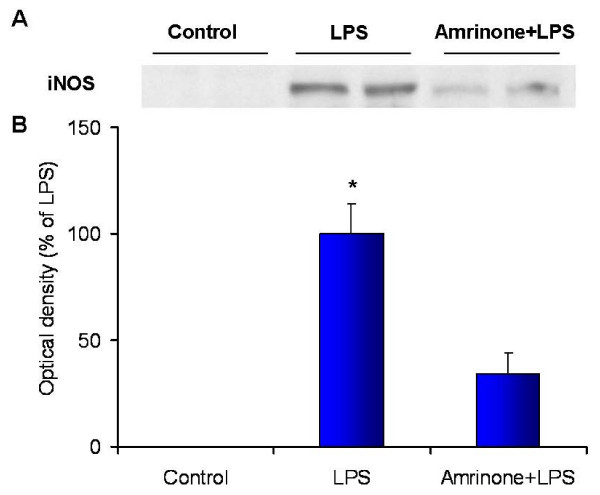
**Endotoxin caused increased inducible nitric oxide synthase (iNOS) expression in the kidney tissue**. Amrinone infusion attenuated iNOS expression. (A) A representative immunoblotting for iNOS in the kidney tissue extracts prepared from control, LPS, and Amrinone+LPS groups (n = 6 per group). (B) Densitometric results are expressed as a percentage compared with the mean value in LPS group (100%). *P < 0.01 versus Control or Amrinone+LPS.

Administration of LPS significantly increased iNOS protein in the kidney, while the PDE3 inhibitor significantly attenuated iNOS protein expression (Figure 7). iNOS was detected at 130 kD in the kidney tissue from LPS and to a lesser degree in the amrinone+LPS groups. These results suggested that there may be an association between PDE3 inhibition and iNOS down regulation.

## Discussion

In the present study, we investigated the role of PDE3 in acute kidney injury induced by endotoxin. Increased expression of PDE3B along with decreased cAMP in the exdotoxemic kidneys were associated with reduced renal function, alteration of renal tubular cell morphology, and increased iNOS expression. The present data suggested that the PDE3 inhibitor, amrinone, may have a protective role in endotoxemic rats with restoration of tissue levels of cAMP and reduction of iNOS expression, associated with improved renal function and morphology.

PDE3A is highly expressed in the cardiovascular system, whereas PDE3B is expressed in adipocytes and hepatocytes [10]. In the present study, PDE3B was expressed only in the distal tubule in the control group, and it was highly expressed in the proximal tubule when endotoxin was administered. However PDE3A was not detected in the kidney in either control or LPS pretreated groups. Although amrinone is a selective PDE3 enzyme inhibitor, it does not discriminate between PDE3A and PDE3B [8]. We only found PDE3B in the kidney, and thus presumably amrinone was inhibiting only PDE3B, suggesting that PDE3B may have a role in endotoxemic kidney injury.

Lipopolysaccharide (LPS) can cause a decrease in tissue cAMP concentration [4]. The present study also showed decreased cAMP concentration in the kidney tissue after endotoxemia and it was preserved by an infusion of amrinone. An increased tissue cAMP level has been shown to protect against hypoxemia induced endothelial injury by preserving endothelial barrier function [5], to improve post-ischemic recovery in the liver and kidney [6,7], and to regulate mitochondrial function [28,29]. Increasing cAMP attenuates iNOS expression [30]. Therefore, preserved cAMP by the PDE3 inhibitor may attenuate iNOS expression which in turn may reduce endotoxin-induced kidney injury.

The pathophysiology of sepsis-induced acute renal failure is still under investigation. Several mediators such as activated protein C, Caspase 1, Caspase 3, and ICAM-1 do play a pivotal role in animal models of sepsis-induced acute kidney injury [31-34]. Activated protein C and caspase 3 were linked to iNOS in a septic kidney model [31,32]. Sepsis causes vasodilation in the systemic circulation that is mediated by iNOS activation [35]. Peritubular capillary dysfunction has a significant role in renal cortical perfusion during endotoxemia [36]. Inhibition of iNOS by specific iNOS inhibitor has been shown to preserve renal cortical perfusion during endotoxemia [31]. Futhermore, upregulation of renal inducible nitric oxide synthase during human endotoxemia and sepsis is associated with proximal tubule injury [37]. The generation of oxygen radicals during sepsis may cause peroxynitrite-related tubular injury [3]. In the present study, the PDE3 inhibitor, amrinone, raised tissue levels of cAMP and attenuated LPS-induced increased renal iNOS protein expression as well as restoring renal function. Specific iNOS inhibitors are effective in attenuating kidney injury with LPS induced renal dysfunction [13,14]. Taken together, downregulation of iNOS by the PDE3 inhibitor may account for attenuated proximal tubule damage and preserved renal function.

Light microscopic examination of renal tissue revealed normal histology (data not shown) similar to the findings of others using high dose of LPS [38,39]. However ultrastructural changes were present in all endotoxemic rat kidneys similar to that shown by others [39-41]. In the proximal tubule, intracellular edema and loss of mitochondria integrity were observed in the LPS pretreated rat kidney. Amrinone infusion attenuated these ultrastructural change as well as restoring serum BUN and creatinine.

In contrast to an earlier study [21], the PDE3 inhibitor is effective at preventing LPS induced kidney injury. The difference between the two studies are LPS dose, whether the rats were anethetized or not, and the use of different types of PDE3 inhibitor. A lower dose of LPS (1 mg/kg) was used in the present study compared to the other (4 mg/kg). Rats were anesthetized in the present study which may account for the lower baseline MAP and and anesthesia may also lead to less activation of sympathetic nerve system resulting in less inflammation [42,43]. Furthermore the PDE3 inhibitor, milrinone, was used in the former study and it is known to have less anti-inflammatory effects [15,21]. Taken together, all of above factors may contribute conflicting results with the previous study.

Increased cAMP increases muscle contractions in myocardium and relaxation of vascular smooth muscle [44], so amrinone may lead to lowering of blood pressure. Although LPS did not cause significant hypotension throughout the experiment, amrinone significantly reduced MAP (Fig. 1). However, MAP remained above 80 mm Hg throughout the experiment in the amrinone group. Therefore the amrinone dose in our study inhibited PDE3 without serious hemodynamic changes. Amrinone inhibits both PDE3A and PDE3B. PDE3A is present in cardiac muscle and vascular smooth muscle [45] but was not detectable by us in the kidney, and Amrinone had little or no effect on kidney function in control rats [46]. PDE3B which is an important regulator of insulin secretory processes and metabolism [47] was found by us in the kidney. Therefore, the combined positive inotropic and vasodilating property of amrinone are due to inhibition of PDE3A. Thus attenuation of LPS induced acute kidney injury was likely accomplished by inhibition of PDE3B. Furthermore, amrinone has significant anti-inflammatory activity independent of its hemodynamic effects [15,23].

## Conclusion

In this study, LPS caused a decrease in renal tissue cAMP that was associated with an increased expression of PDE3B, but not PDE3A. The PDE3 inhibitor, amrinone, averted the fall in tissue cAMP levels, prevented the rise in iNOS expression, and attenuated the renal tubular injury induced by LPS. Taken together, it is suggested that PDE3B inhibition may have a clinical potential in the treatment of sepsis-induced acute kidney injury.

## Abbreviations

ARF: Acute renal failure; ANOVA: Analysis of variance; BUN: blood Urea Nitrogen; cAMP: cyclic-3',5'-adenosine monophosphate; GAPDH: glyceraldehyde-3-phosphate dehydrogenase; H_2_O_2_: hydrogen peroxide; iNOS: inducible nitric oxide synthase; LPS: lipopolysaccharide; MAP: mean arterial pressure; PBS: phosphate buffered saline; PDE: phosphodiesterase; RT-PCR: reverse transcription-polymerase chain reaction.

## Competing interests

The authors declare that they have no competing interests.

## Authors' contributions

WIC was responsible for carrying out the experiments, for data analysis, and for drafted this manuscript; KYK and JWS was responsible for the analysis and design for the histologic study; JB oversaw the animal experiments, instructed WIC in his implementation; DAQ and CAH is an expert in sepsis experiment and assisted in the experimental design and the data analysis and interpretation. All authors contributed to the drafting and revisions of the manuscript.

## Pre-publication history

The pre-publication history for this paper can be accessed here:

http://www.biomedcentral.com/1471-2334/9/80/prepub
